# Protocol for the economic evaluation of integrated community-based care compared with integrated facility-based care for HIV, hypertension and diabetes in Tanzania and Uganda (INTE-COMM trial)

**DOI:** 10.3310/nihropenres.13794.2

**Published:** 2025-01-29

**Authors:** Gerard Joseph Abou Jaoude, Ivan Namakoola, Faith Aikaeli, Godfather Kimaro, Faith Moyo, Francis Xavier Kasujja, Erik Van Widenfelt, Sokoine Kivuyo, Josephine Birungi, Gerald Mutungi, Omary Said Ubuguyu, Stephen Watiti, Kaushik Ramaiya, Sayoki Mfinanga, Moffat Nyirenda, Anupam Garrib, Shabbar Jaffar, Jolene Skordis, Neha Batura

**Affiliations:** 1 University College London, Institute for Global Health, London, England, WC1N 1EH, UK; 2 Non Communicable Diseases Research, MRC/UVRI and LSHTM Uganda Research Unit, Entebbe, Central Region, Uganda; 3 National Institute for Medical Research Muhimbili Research Centre, Dar es Salaam, Dar es Salaam, Tanzania; 4 Non Communicable Diseases Control Program, Ministry of Health, Kampala, Kampala, P.O.Box 7272, Uganda; 5 Non Communicable Diseases, Ministry of Health Community Development, Dodoma, P.O.Box 743, Tanzania; 6 Community Health Alliance, Kampala, P.O.Box 22205, Uganda; 7 CEO, Shree Hindu Mandal Hospital, Dar es Salaam, Dar es Salaam, Tanzania

**Keywords:** Economic evaluation, HIV, diabetes, hypertension, non-communicable diseases, integrated care, community care, sub-Saharan Africa

## Abstract

**Background:**

The number of people living with multiple chronic conditions in sub-Saharan Africa is increasing, but health facilities are unable to meet demand. To improve health system capacity and access to care, community models of HIV care have been trialled in countries such as Tanzania and Uganda. However, no evidence exists to inform policymakers on the effectiveness and cost-effectiveness of integrated community-based models of care for HIV and chronic non-communicable conditions. This protocol outlines a within-trial economic evaluation to address this gap.

**Methods & analysis:**

We will estimate the costs and cost-effectiveness of integrated community-based care for HIV, hypertension and diabetes compared with facility-based care within the INTE-COMM pragmatic cluster-randomised trial in Tanzania and Uganda. Analyses will adopt a 52-week time horizon, the duration of trial follow-up. The full enrolled trial sample will be analysed from a societal perspective, comprising provider and patient perspectives. Economic costs will be estimated, which includes valuing inputs such as donated goods or time foregone by participants because of receiving care. For provider costs, participant case report forms will inform resource use along with data from facilities and community sites. Resources will be valued using project accounts, facility spending, and locally available cost data. Patient costs will be estimated based on a care-seeking and cost questionnaire administered to participants. Estimated costs will be analysed with co-primary trial outcomes on plasma viral load suppression, glycaemia and blood pressure control to calculate incremental cost-effectiveness ratios (ICER). We will also calculate ICERs for secondary trial outcomes related to health-related quality of life and wellbeing. Cost drivers and outcomes will be varied within confidence bounds in a two-way sensitivity analysis. We will investigate equity impact by estimating the mean difference in outcomes between integrated community-based and facility-based care across household socio-economic quintiles and by measuring whether participants incurred catastrophic health expenditures.

**Trial registration number:**

The ISRCTN Registry: ISRCTN15319595. Registered on 07 June 2022: https://doi.org/10.1186/ISRCTN15319595

## Plain Language Summary

Our study aims to evaluate the type of HIV, diabetes and hypertension care which offers the best value for money in sub-Saharan African settings. This will involve comparing the costs and benefits among patients attending integrated community-based or facility-based care in Tanzania and Uganda. Unlike usual care, where patients attend separate clinics for different health conditions, integrated care means patients receive care in one place by the same health workers regardless of their health condition. The study will be carried out across community sites, primary healthcare clinics and hospitals in both countries.

Costs will be collected and estimated from different perspectives. The costs to healthcare providers will be estimated separately for facilities and community sites. Alongside provider costs, the study will evaluate the financial burden incurred by patients from illness and seeking care in community sites and facilities. To estimate benefits to patients receiving community-based or facility-based care, we will capture outcome changes specific to each health condition in addition to changes in quality of life and wellbeing.

Policymakers are faced with difficult decisions on how to use limited resources to improve social welfare. Ultimately, our study seeks to inform policymakers in Tanzania, Uganda and sub-Saharan Africa more widely on how to allocate resources for HIV, diabetes and hypertension care based on the options available to them.

## Introduction

Recent estimates suggest that over a quarter of the adult population in sub-Saharan Africa (SSA) is living with hypertension ^1^ and 4.5% with diabetes ^2^. However, despite the increasing prevalence of these chronic conditions, only a small proportion of people with hypertension or diabetes are in regular care, and those who are tend to have suboptimal health outcomes ^3– 5^. In contrast, SSA has high coverage of antiretroviral therapy provision and HIV viral suppression ^6^ through health facilities as well as in community settings ^7, 8^.

Health services in SSA and other low- and middle-income countries are often provided vertically. This can lead to inefficiencies in healthcare provision due to duplication of services ^9, 10^ and can add to the economic burden borne by patients with multiple chronic diseases, who must attend several clinics to receive condition-specific care ^11, 12^. Recent evidence from the INTE-AFRICA trial showed that a model of integrated care (compared with vertical care) for patients with HIV, hypertension, and/or diabetes in primary care facilities in Tanzania and Uganda was acceptable and resulted in high retention in care, equivalent clinical outcomes, and cost-savings from the provider perspective ^9, 10, 13– 16^.

However, facility-based care remains constrained by the shortage of qualified health workers, available infrastructure, and can pose high direct and indirect costs to individuals seeking care which in turn may result in lower patient adherence to care for chronic conditions such as hypertension and diabetes ^17^. There are therefore substantial challenges hindering the scale up of chronic care provision, solely through facility-based care, to levels that will meet existing and future population health needs. As experienced in the case of HIV in SSA, a feasible solution could be to provide care in the community ^18^, which facilitates the scale up of service provision through a reduced reliance on existing infrastructure ^19^.

Community-based HIV testing and linkage to care interventions, compared with facility-based provision, have been shown to be acceptable and to improve rates of detection, linkage to care and viral load suppression ^19– 21^. Alongside this, economic evidence has shown that community-based HIV services can be cost-effective in SSA settings ^22– 24^, although results vary as expected depending on the type of intervention, underlying prevalence, and whether key populations are targeted. However, evidence on the value for money of integrated community-based care for HIV and non-communicable conditions in SSA is limited ^17, 25, 26^.

While some partial evaluations provide evidence on the cost of integrated screening programmes for HIV and non-communicable conditions in SSA ^25, 26^, to the best of our knowledge, there is no economic evaluation of an integrated community-based model of comprehensive care. Yet assessments of cost-effectiveness and affordability are key to inform effective policymaking and resource allocation. The proposed economic evaluation in this protocol, embedded within the INTE-COMM trial, will fill this evidence gap by estimating the costs and cost-effectiveness of integrated community-based care compared with integrated facility-based care for people with HIV, diabetes and/or hypertension.

### The INTE-COMM trial

INTE-COMM is a pragmatic cluster-randomised trial of an integrated community-based model of care for HIV, hypertension and/or diabetes compared with an integrated facility-based model of care in Tanzania and Uganda ^18^. Groups of participants in each country have been randomised to integrated community-based and facility-based care according to a 1:1 ratio. Participants were clustered into groups based on their residence and nearby health facility, and then randomly assigned to different trial arms. Participant groups in the community-based arm then went on to receive care at a local community venue rather than the health facility. The duration of trial follow-up is 12 months after groups initially meet at their health facility. Participants in the community-based arm have been meeting monthly, while participants in the facility-based arm have attended according to their usual visitation schedule. More detail on INTE-COMM can be found in the main trial protocol ^18^. The sections below outline the health economic analysis plan for the within-trial economic evaluation.

### Objectives

We will carry out a within-trial economic evaluation of integrated community-based care for HIV, hypertension and/or diabetes compared with integrated facility-based care from a provider, patient and societal (sum of provider and patient) perspective to estimate the:

1. Average cost per person receiving community-based and facility-based care;
2. Incremental cost and cost-effectiveness of community-based care compared with facility-based care;
3. Total cost of implementing community-based care at scale compared with facility-based care; and
4. Equity impact of benefits experienced and costs incurred by participants.

Provider perspective results will inform policymakers on the value for money of community-based care and its affordability based on available public funds to support evidence-based resource allocation that maximises population health outcomes. Patient and societal perspective results will also inform decision-makers on how community-based care affects costs borne by patients from seeking care, relative to a facility-based alternative.

## Methods

### Patient and Public Involvement

The baseline participant and household questionnaire, participant care-seeking and cost questionnaire, as well as ICECAP-A secondary outcome questionnaire were tested with a group of participants before being employed for wider data collection. Feedback from participants on the duration, acceptability and understanding of the questionnaires informed the final versions used with the full trial sample. No participants or members of the public will be involved in conceptualising other aspects of the economic evaluation study, methods or data analysis. However, feedback from decision-makers and other relevant study stakeholders will inform how results are presented to maximise their potential uptake by policymakers. A separate process evaluation study will also provide greater insight into the experience of participation in the trial.

The INTE-COMM trial more widely is a project of the RESPOND-AFRICA group, which includes a Patient and Public Involvement (PPI) group of care providers, facility managers, community stakeholders, health workers, patient representatives, policymakers, non-governmental and civil society organisations. The PPI group has been engaged throughout the study cycle and contributed to the design of INTE-COMM. The PPI group is also instrumental for the ongoing evaluation of INTE-COMM, having contributed to key aspects such as choices of primary and secondary measures. Engagement with the PPI group will continue and will include other key upcoming activities such as dissemination. More information on PPI in INTE-COMM is detailed in the main trial protocol ^18^.

### Study setting and population

The INTE-COMM study is being carried out in two types of sites. The control (facility-based) arm comprises six government health facilities in Tanzania and eight in Uganda. The health facilities are primarily in urban and peri-urban settings, consisting of hospitals in Tanzania and a mix of hospitals and primary clinics in Uganda. All the facilities have on-site pharmacies and laboratories, and most have inpatient beds. Each community venue and group of participants within a venue is associated with a participating health facility. Community venues in the intervention (community-based) arm consist of a wide variety of sites, ranging from community leader’s home compounds to spaces within religious venues, community halls, schools, local government offices and other public or private spaces.

People aged 18 years or above living with HIV, hypertension and/or type 2 diabetes attending one of the fourteen facilities were eligible for enrolment in the trial. People eligible for recruitment had to be willing to participate in community care, were receiving care for 6 months or more in the facility, were considered adherent to their treatment in the preceding 6 months, and planned to stay in the facility catchment area for 6 months or more. Included participants also had to be on the same treatment regimen for at least 3 months, did not have unmanaged complications/co-infections, and did not require change in clinical management. Pregnant women, participants with blood pressure >160/100mmHg during recruitment and >180/110mmHg more than once in the preceding 6 months, and participants with fasting glycemia >13mmol/L at any time in the previous 6 months were excluded from the trial. People with unmanaged complications or conditions that required care in facility settings were also excluded.

The research team, facility staff and lay health workers (community health workers in Tanzania or village health teams in Uganda) worked together to cluster people who consented to participate in the trial into groups of 12–20 (median: 15) people in Tanzania and 11–19 (median: 15) people in Uganda. This was done based on where people lived in relation to the health facility and their health conditions. Groups were formed with a 2:1 ratio of people living with hypertension and/or diabetes to people living with HIV. Participants were recruited by reviewing their patient records as they came in for their scheduled facility visit and the clinician confirming that they are clinically stable. Participants in a group were recruited on a rolling basis until the target ratio of health conditions and average number of participants were both met. Groups were then randomised to either facility-based or community-based care. A total of 124 groups were recruited across both countries, 59 in Tanzania and 65 in Uganda, with the aim of achieving a total trial sample of around 1736 participants.

### Integrated community-based and facility-based care

The integrated community-based care model was developed through an initial scoping review, followed by government policy reviews to identify community-based HIV care models that could serve as templates, and workshops with key stakeholders. INTE-COMM participants in the intervention arm (or community-based arm) were assigned in groups and had their first group meeting at their respective health facilities. During their first meeting, led by a nurse and a trained community health worker in Tanzania or member of the village health team in Uganda, the group selected their group leader and discussed potential venues in their community for subsequent meetings and care provision. Group leaders, nurses and trained lay health workers then visited and selected suitable venues, before mapping and engaging relevant local stakeholders. Once a venue was established, participants were expected to attend monthly group meetings.

Ahead of each community-based monthly meeting, nurses and lay health workers prepared patient files and the nurse picked-up medications from facility pharmacists as prescribed by health facility clinicians. During the community-based monthly meetings, participants received health education, behavioural and adherence information and support from the lay health worker under nurse supervision in a group setting. Following this, participants individually had their blood sugar and pressure monitored and nurses clinically reviewed participants for progress, as well as to identify needs for referrals to the facility for additional care. Participants then collected their medicines and could leave. If participants missed a monthly visit, in Uganda they were called by phone by a nurse and in Tanzania by a nurse or experienced community health worker, advised to collect their medicines from their health facility and were informed of their next monthly community meeting. The final community-based monthly meetings, or trial endline, are taking place at the health facility.

Lay health workers in the community arm received comprehensive training before the trial, including on the study protocol, communication skills and concepts related to HIV, hypertension and type 2 diabetes. This was followed by refresher and continuous hands-on trainings provided by study nurses. Clinical staff in both arms of the trial received training on how to manage HIV, diabetes and hypertension, along with regular on-the-job training. More information on staff training, monitoring and supervision of intervention and control arm activities can be found in the main trial protocol ^18^.

The control arm (or facility-based arm) in INTE-COMM consists of care in integrated clinics at the health facilities led by clinicians, based on the model of care previously tested in the INTE-AFRICA trial ^9^. In the control arm, INTE-COMM participants living with HIV, hypertension, diabetes or any combination of these conditions shared the same facility registration, triage, waiting, and pharmacy areas. Their records were integrated and they were cared for by the same health workers. Participants in the control arm also received counselling on health education and support for adherence as would be provided in standard or routine facility-based care in each country. The frequency of participant visits in the control arm varied based on individual patient schedules, which are established with clinicians based on their health conditions and clinical status.

### Outcome measurement

The INTE-COMM trial measures two co-primary outcomes ^18^: (1) plasma viral load suppression, defined as less than 1000 copies per ml or an undetectable viral load, and (2) a composite of glycemia and blood pressure control (<7.0mmol/l and <140/90 mmHg). Secondary trial outcomes include general health-related quality of life, measured by the self-reported EuroQoL-5 Dimensions (EQ-5D-3L) questionnaire administered to participants ^27^, and general wellbeing through the self-reported ICEpop CAPability measure for Adults (ICECAP-A) ^28^. More information on trial outcomes can be found in the INTE-COMM trial protocol ^18^.

### Identification, measurement and valuation of resource use

In line with the economic evaluation perspective, cost data is being collected for both service providers and participating patients separately in each trial arm. Data collection is considering direct and indirect costs. Table 1 outlines categories of costs and their respective data sources. Economic costs will be analysed over a 12-month time horizon, based on the trial duration from enrolment to endline.

**Table 1.** Cost categories and data sources.

Description	Type of cost	Data source	Sample size
**Provider costs**			
Cost of adapting and implementing community-based care and facility- based care	Direct	1. Implementing agency project accounts 2. Interviews with project staff 3. Facility-based cost capture tool drawing on facility records, visits, interviews and observations 4. Community-based cost capture tool focused on venue characteristics and floor size 5. Implementing agency project records 6. Participant CRFs	1- 5. n/a 6. Full trial sample
	Indirect	1. Project records on volunteer involvement and donated goods 2. Facility-based cost capture tool drawing on facility records, visits, interviews and observations 3. Interviews with project staff	1-3. n/a
**Patients**			
Cost of health seeking for patients and their households	Direct	1. Direct medical cost of care-seeking as well as related transport and food cost (combined as “travel costs”), self-reported in the patient careseeking and cost questionnaire. 2. Participant case report forms	1. All participants from the endline survey (c. 50% of the trial sample) 2. Full trial sample
Opportunity cost of participating in community-based care and facility- based care	Indirect	1. Lost productivity due to care-seeking, self-reported in the patient careseeking and cost questionnaire. 2. Participant case report forms	1. All participants from the endline survey (c. 50% of the trial sample) 2. Full trial sample

A mix of bottom-up and top-down costing approaches will be adopted to estimate provider costs for the community-based and facility-based trial arms. For example, the cost of facility overheads in the facility-based trial arm will likely be estimated from a top-down approach by allocating high-level expenditures to individual patients. In contrast, medicine costs for both trial arms will likely be estimated using a bottom-up approach, based on the volumes of medicine units and dosages used by participants combined with the respective medicine prices per unit. To estimate the one-off costs of developing and setting up the community-based model of care, as well as some of the implementation costs in both trial arms, data are being extracted from partner project accounts and separated into set-up or implementation activities. The cost of any donated goods or volunteer time captured in project records or interviews, for both trial arms, will be estimated based on the closest equivalent current market value.

For the facility-based arm provider implementation costs, data on service resource use and visit frequencies will be acquired from participant case report forms (CRFs) and facility data. Service and resource use will then be valued based on a facility cost capture tool which has been used to collect data from representative urban and rural health facilities in Uganda (n=4) and Tanzania (n=3). The facility cost capture tool covered facility characteristics, the type and number of services provided, expenditures, staff salaries and time allocations, general and INTE-COMM specific facility surface areas, pharmacy and laboratory activities and prices, capital costs (including vehicles), furniture and equipment, donated goods or volunteer time, and consumables.

For the community-based arm provider implementation costs, data on service resource use and visit frequencies will also be captured from CRFs. A community-based cost capture tool was used to collect data on characteristics and floor sizes from each community site in both countries. We will estimate the closest equivalent current market value for the use of community sites based on the local rental costs per square meter for comparable venues.

Costs incurred by participants will be estimated based on data collected through a questionnaire administered to participants between six and twelve months of being enrolled on the trial. The questionnaire asks about their visit on the day and about any health problems or careseeking in the preceding three months. This includes information on travel time and costs, medical service use and costs (consultation, medicines, diagnostic), referrals, any other outpatient or inpatient use, how costs were paid for, and opportunity costs of participating – i.e. time for other activities foregone because of receiving care.

Costs will be captured and presented by trial arm, separately for Tanzania and Uganda. Price years and currencies of cost data will be recorded, before adjusting for inflation to a base year (2023) using the respective consumer price indices for Tanzania and Uganda. To enable comparability between countries and with other studies, all costs will be converted and presented in international dollars in addition to local currencies. As the time horizon is 12-months, discounting will only be applicable to capital goods, which have lifespans of several years. Capital costs will be annuitized using annual rates of 3% in the main analysis and values of 0%, 6% and government bank bond yields in sensitivity analyses ^29^.

### Economic evaluation

To inform decision-makers on the value for money of integrated community-based care relative to its facility-based alternative, a cost-effectiveness analysis will be carried out based on the full enrolled trial sample, regardless of duration on the trial. The analysis will be reported according to the Consolidated Health Economic Evaluation Reporting Standards (2022) and where applicable will draw on best-practice guidance in reference cases for economic evaluations ^30, 31^. Main analysis results will include the average cost per community-based and facility-based participant, as well as combine costs and outcomes in incremental cost-effectiveness ratios (ICERs) of community-based care relative to facility-based care. ICERs will be the arithmetic mean difference in cost between community-based care and facility-based care divided by the arithmetic mean difference in effect based on trial outcomes.

The co-primary trial outcome of plasma viral load suppression will inform the ICER expressed as an incremental cost per additional virally suppressed participant at endline (12 months). The other co-primary trial outcomes, glycaemia and blood pressure control, will inform ICERs similarly expressed as an incremental cost per additional participant with controlled glycemia or blood pressure at endline. However, the co-primary trial outcomes are not typically used in other economic evaluations (particularly for hypertension and diabetes), do not enable comparisons between interventions and studies, nor capture broader intervention effects on health-related quality of life. To address this, quality-adjusted life years (QALYs) will be calculated based on participant responses to the self-reported EQ-5D-3L questionnaire and used to estimate an ICER expressed as an incremental cost per QALY gained.

It is possible that community-based care impacts more broadly on participant wellbeing (e.g. through reduced stigma and challenges of attending facility-based care or through the support provided by the group), which would not be captured by the EQ-5D and QALYs. To investigate this, years of full capability (YFC) ^32^ will also be estimated based on participant responses to the self-reported ICECAP-A questionnaire, which measures general wellbeing based on Sen’s capability approach. The ICER based on the ICECAP-A will be presented as an incremental cost per YFC.

The different ICERs estimated will be presented alongside each other. Univariate and two-way sensitivity analyses will investigate the impact on results of varying outcome effect sizes within confidence bounds and key cost drivers, such as staff or medicines for provider costs and travel or medical costs for participants. If intervention effects on health-related quality of life and wellbeing are positive, statistically significant, and sustained throughout the duration of trial follow-up, we will explore the possibility of employing a longer economic evaluation time horizon (e.g. 10-years) through decision-analytic modelling.

While interventions can be cost-effective relative to alternatives, a key consideration for policymakers and implementers is the affordability and feasibility of available options. The INTE-COMM economic evaluation will therefore estimate the total cost to providers of scaling up community-based care compared with facility-based care. Total costs will be estimated based on national prevalence rates of HIV, hypertension and diabetes, separately for Tanzania and Uganda. To investigate affordability, the estimated total cost of implementing community-based care and facility-based care at scale in each country will then be expressed as a percentage of available national health spending from public sources and gross domestic product.

### Equity impact

We will investigate how outcomes of integrated community-based care are distributed across population groups based on socio-economic status, to see whether some population groups benefit disproportionately from the intervention compared with facility-based care. The equity impact of the INTE-COMM trial will be assessed by estimating the marginal mean difference in outcomes of community-based care compared with facility-based care within socioeconomic status quintiles. Quintiles will be estimated based on participant responses to a baseline questionnaire. The questionnaire includes information on participant and other household member characteristics, household characteristics, assets, income and self-assessed financial status. Socioeconomic quintiles will be constructed based on: (1) income or a composite asset index, and (2) a multidimensional index which considers both monetary and non-monetary dimensions of deprivation. The equity analysis will also investigate whether costs incurred by participants caused them financial hardship, measured through catastrophic health expenditures. Catastrophic health expenditure is typically defined as patient costs exceeding a specified proportion of their total income, expenditure or consumption or their capacity to pay (non-essential spending or consumption). In this economic evaluation, household income adjusted to a per capita amount will be used, based on participant responses to the baseline questionnaire. Catastrophic health expenditures will be estimated by income quintiles if the final sample size allows.

## Discussion

The full economic evaluation described in this protocol will be the first in sub-Saharan Africa to evaluate the cost and cost-effectiveness of integrated community-based care compared with facility-based care for adults living with HIV, hypertension and/or diabetes. Results from this study can inform the prioritisation of integrated care models in sub-Saharan Africa. The data collection and analysis activities outlined in the protocol enable transparency and comparison with other studies. Results will be presented separately for Tanzania and Uganda, to inform policymakers in each country, and will be disaggregated to improve the potential for transferability to other settings. Economic evaluation results will be disseminated to various target audiences in the form of peer-reviewed journal publications, policy briefs, conferences, workshops and meetings with national and/or international stakeholders.

### Ethics and dissemination

The INTE-COMM trial and economic evaluation received written ethical approval from the National Institute of Medical Research in Tanzania (NIMR/HQ/R.8a/Vol. IX/3977 (28 ^th^ April 2022)), the Uganda Virus Research Institute (GC/127/872 (23 ^rd^ March 2022)), the Uganda National Council for Science and Technology (HS2278TS (13 ^th^ July 2022)), the London School of Hygiene and Tropical Medicine Ethics Committee (28122 (31 ^st^ August 2022)) and the University College of London (2382/001 (25 ^th^ October 2022)).

Before data collection, delegated research staff read and explained the INTE-COMM study information sheet to participants in Swahili or Luganda and answered participant questions. Participants were informed about the study objectives, procedures, and the risks and benefits of participation. The information sheet also emphasised that participation in INTE-COMM is entirely voluntary and that participants can withdraw at any time without compromising the standard care they receive at the study site. For individuals that agreed to participate, the participant and research staff wrote their full name, dated, and signed the consent form. As approved by the ethics committees, if individuals could not read or write, oral consent was accepted in the presence of a witness that was not part of the health personnel at the trial site and the signature of the witness was collected. The information sheet and a copy of the signed informed consent form were given to participants. The dates of the information and the signature of the consent form were recorded in the electronic case report form database with the name of the research staff who obtained the informed consent.

Findings from the economic evaluation will be disseminated in English, and local languages when necessary, to the scientific community, policymakers and wider public through: (1) peer-reviewed publications, (2) policy briefs, workshops and webinars, (3) conferences, and (4) accessible materials on the INTE-COMM project webpages and social media.

**Contributors:** GAJ, NB, and JS conceptualised the economic evaluation study design. GAJ developed the data collection tools and analysis plan, with support from NB. GAJ wrote the first draft, reviewed by NB and JS. All authors reviewed and contributed to subsequent drafts. SJ, SM and MN acquired funding for the INTE-COMM trial.

## Data Availability

No data are associated with this article.
