# Supplementary material for: Protocol for the economic evaluation of integrated community-based care compared with integrated facility-based care for HIV, hypertension and diabetes in Tanzania and Uganda (INTE-COMM trial)
Source: NIHR Open Res. 2025 Jan 29;4:80. Originally published 2024 Dec 24. [Version 2] doi: 10.3310/nihropenres.13794.2 (PMC11868744; doi:10.3310/nihropenres.13794.2)
Supplement: Peer review history

# Reviewer report: Reviewer response for version 2

**DOI:** 10.3310/nihropenres.15092.r34716

**Published:** 2025-03-03

**Authors:** Lise Jamieson, Nkgomeleng Lekodeba

**Affiliations:** 1 Health Economics and Epidemiology Research Office, University of Witwatersrand, Johannesburg, South Africa

The protocol outlines an economic evaluation within the INTE-COMM trial, a cluster-randomized study comparing integrated community-based and facility-based care for HIV, hypertension, and/or diabetes in Tanzania and Uganda. The evaluation will estimate costs and cost-effectiveness from both provider and patient perspectives. Primary outcomes include viral load suppression, glycemia, and blood pressure control. The study also considers health-related quality of life (QALYs) based on a self-reported EQ-5D-3L questionnaire conducted within the trial. Cost analyses will use both bottom-up and top-down approaches, with sensitivity analyses planned. 

1. Could the authors expand on how they would incorporate non-monetary dimensions of deprivation into constructing socioeconomic quintiles? I think in general just more detail on how these quintiles will be constructed would be useful.
2. Many studies define the societal perspective as simply including both provider and patient costs, but a true societal perspective should go beyond that. It should include all costs and impacts on society, including productivity losses to the economy due to illness or spending time at facilities accessing care, caregiver burden, and other spillover effects: onward HIV transmission impacts, reduced burden on social support programs, and so on. If the authors want to truly claim a societal perspective, they should either expand the analysis to include productivity losses and broader externalities or clearly state that they are using a limited societal perspective, rather than a full one.
3. Some costing methods and sources could be elaborated on a bit more.
  1. The protocol states that training was provided to community-based health workers and clinical staff, but it does not specify the cost breakdown. Were costs estimated per trainee, per session, or as a lump sum? Additionally, were opportunity costs (e.g., time that health workers spent in training instead of delivering care) factored into the cost calculations? What is the source of the cost of training?
  2. The economic evaluation should explicitly detail how staff time was estimated – was this based on self-report by staff or was a time-in-motion study conducted? How were differences in workload between facility-based and community-based care accounted for across different staff types.
  3. Additionally, community-based models typically require additional supervision to ensure fidelity to protocols. Were these costs estimated and included in the provider cost calculations? If supervision costs are covered within existing health system budgets, was this factored into the interpretation of cost-effectiveness?
  4. The rationale for selecting a 12-month time horizon is not explicitly stated, aside from its relevance to discounting capital goods. Providing further justification for this choice would enhance transparency.
  5. Given that the study includes data from two countries, specifying the exchange rates and base currency used for conversion to international dollars would improve clarity.
  6. While the economic costs are well outlined in the protocol, I think the authors can also distinguish what constitute financial costs. It appears to me that they will also be used for cost estimation, the actual prices of goods and services but not much attention is directed to specifying what such cost entails.
4. Other minor things:
  1. The abstract specifies a time horizon of 52 weeks, while the main text refers to 12 months. Although these are equivalent, maintaining consistency in wording throughout the document would improve clarity.
  2. On page 7, paragraph 4, line 2, the text is written in past tense. Should this be in future tense, given that data collection and analysis are ongoing?

Is the study design appropriate for the research question?

Yes

Is the rationale for, and objectives of, the study clearly described?

Yes

Are sufficient details of the methods provided to allow replication by others?

Partly

Are the datasets clearly presented in a useable and accessible format?

Not applicable

Reviewer Expertise:

HIV, health economics

We confirm that we have read this submission and believe that we have an appropriate level of expertise to confirm that it is of an acceptable scientific standard.

# Reviewer report: Reviewer response for version 2

**DOI:** 10.3310/nihropenres.15092.r34656

**Published:** 2025-02-27

**Authors:** Callistus Akinleye

**Affiliations:** 1 Osun State University, Osogbo, Nigeria

The conclusions supported by the data presented.

 The limitations of the  Protocol and evaluation were clearly described.

 The authors discussed how these intervention can be helpful to advance our understanding of the topic under study.

 Great public health intervention relevance addressed.

 The economic evaluation presented matches the analysis plan.

 The Protocol is clearly and completely presented.

 The Table is of sufficient quality for clarity.

Is the study design appropriate for the research question?

Yes

Is the rationale for, and objectives of, the study clearly described?

Yes

Are sufficient details of the methods provided to allow replication by others?

Yes

Are the datasets clearly presented in a useable and accessible format?

Yes

Reviewer Expertise:

Genomics, Bioinformatics, NCDs, HIV/TB, MDR TB

I confirm that I have read this submission and believe that I have an appropriate level of expertise to confirm that it is of an acceptable scientific standard.

# Reviewer report: Reviewer response for version 2

**DOI:** 10.3310/nihropenres.15092.r34647

**Published:** 2025-02-12

**Authors:** Salma Abdi Mahmoud

**Affiliations:** 1 The State University of Zanzibar, Zanzibar, Tanzania

I have reviewed the protocol, and I find it suitable for acceptance. Please proceed with the necessary steps for indexing.

Is the study design appropriate for the research question?

Yes

Is the rationale for, and objectives of, the study clearly described?

Yes

Are sufficient details of the methods provided to allow replication by others?

Yes

Are the datasets clearly presented in a useable and accessible format?

Yes

Reviewer Expertise:

MEDICINE

I confirm that I have read this submission and believe that I have an appropriate level of expertise to confirm that it is of an acceptable scientific standard.

# Reviewer report: Reviewer response for version 1

**DOI:** 10.3310/nihropenres.14983.r34424

**Published:** 2025-01-23

**Authors:** Salma Abdi Mahmoud

**Affiliations:** 1 The State University of Zanzibar, Zanzibar, Tanzania

Methods

 Assessment: Partly.

 The methods section provides a solid foundation but lacks certain critical details that are necessary for replication by other researchers. Constructive Feedback:

 To improve scientific rigor and replicability, the authors should:

- Provide more detailed information about the baseline characteristics of the selected populations, including socioeconomic and geographic diversity.
- Elaborate on the methods for measuring equity impacts, particularly in quantifying outcomes across different socioeconomic groups.
- Describe any training provided to community-based care providers and the mechanisms for monitoring fidelity to intervention protocols.
- Specify how missing data will be handled in the analysis.

These additions will enhance the clarity and reproducibility of the study methods.

Is the study design appropriate for the research question?

Yes

Is the rationale for, and objectives of, the study clearly described?

Yes

Are sufficient details of the methods provided to allow replication by others?

Yes

Are the datasets clearly presented in a useable and accessible format?

Yes

Reviewer Expertise:

MEDICINE

I confirm that I have read this submission and believe that I have an appropriate level of expertise to confirm that it is of an acceptable scientific standard, however I have significant reservations, as outlined above.

# Response

**Published:** 2025-01-27

**Authors:** Gerard Joseph Abou Jaoude

**Affiliations:** Institute for Global Health, University College London Institute for Global Health, London, England, UK

**We sincerely thank the reviewer for their prompt and helpful feedback on our protocol. We have responded to each of the comments below and specify how and where we have incorporated suggestions in our revised protocol. **

- Reviewer: Provide more detailed information about the baseline characteristics of the selected populations, including socioeconomic and geographic diversity.
  - **RESPONSE: Given that this is a protocol, data analyses have not yet begun. However, baseline characteristics will be analysed and reported within the economic evaluation publication.**
- Reviewer: Elaborate on the methods for measuring equity impacts, particularly in quantifying outcomes across different socioeconomic groups.
  - **RESPONSE: Thank you very much for this and we agree that the wording could be clearer. We have now included more specific wording on our planned approach within the “Equity impact” section and added a sentence explaining the basis on which we plan to construct quintiles. **
- Reviewer: Describe any training provided to community-based care providers and the mechanisms for monitoring fidelity to intervention protocols.
  - **RESPONSE: The main trial protocol contains information on training and intervention monitoring, along with other aspects of the trial. To avoid substantial repetition between the publications, we summarise trial information in this economic evaluation protocol and refer readers to the main trial protocol for additional details. **
  - **However, we agree that information on training is directly relevant to costs captured within the economic evaluation. We have therefore included a summary paragraph within the “Integrated community-based and facility-based care” section to outline how training was carried out for community-based providers.**
- Reviewer: Specify how missing data will be handled in the analysis.
  - **As data analyses have not begun, we do not yet have information on the nature of missing data, and therefore, it is not possible to state how it will be handled. If we find that the data are missing at random, we anticipate handling this via multiple imputation methods. However, if data are not missing at random we will need additional considerations relating to the reasons behind this and potential bias. We may need to employ other imputation approaches such as selection/Heckman model or extensions of likelihood-based methods.**
